# Pharmaceutical Agents for Targeting Autophagy and Their Applications in Clinics

**DOI:** 10.3390/ph17101355

**Published:** 2024-10-11

**Authors:** Ulash Kench, Susanna Sologova, Elena Smolyarchuk, Vladimir Prassolov, Pavel Spirin

**Affiliations:** 1 Department of Cancer Cell Biology, Engelhardt Institute of Molecular Biology, Russian Academy of Sciences, Vavilova 32, 119991 Moscow, Russiaprassolov45@mail.ru (V.P.); 2 Department of Pharmacology, Sechenov University, 119019 Moscow, Russia; 3 Center for Precision Genome Editing and Genetic Technologies for Biomedicine, Engelhardt Institute of Molecular Biology, Russian Academy of Sciences, Vavilova 32, 119991 Moscow, Russia

**Keywords:** autophagy, lysosomes, autophagy modulators, diseases

## Abstract

Autophagy is the process by which damaged regions of the cytoplasm and intracellular pathogens are degraded. This mechanism often serves an adaptive role in cells, enhancing their survival. It plays a direct or indirect role in the development of various pathological conditions within the body. This phenomenon is common in various malignant diseases, where autophagy is associated with the resistance of transformed cells to chemotherapy. Conversely, abnormal activation of autophagy can trigger cell death, a process often seen in neurodegenerative conditions. Given that dysregulation of autophagy is associated with the progression of numerous pathological conditions, this is of significant interest to the developers of drugs that can effectively modulate autophagy for both basic research and clinical applications. Here, we provide a brief description of the mechanism of macroautophagy, the most prevalent form of autophagy identified in humans. We also discuss the clinical potential of drugs that can modulate autophagy, highlighting their use in combating diseases associated with direct or indirect dysregulation of this essential process.

## 1. Introduction

### Mechanism of Autophagy

**Figure 1:**
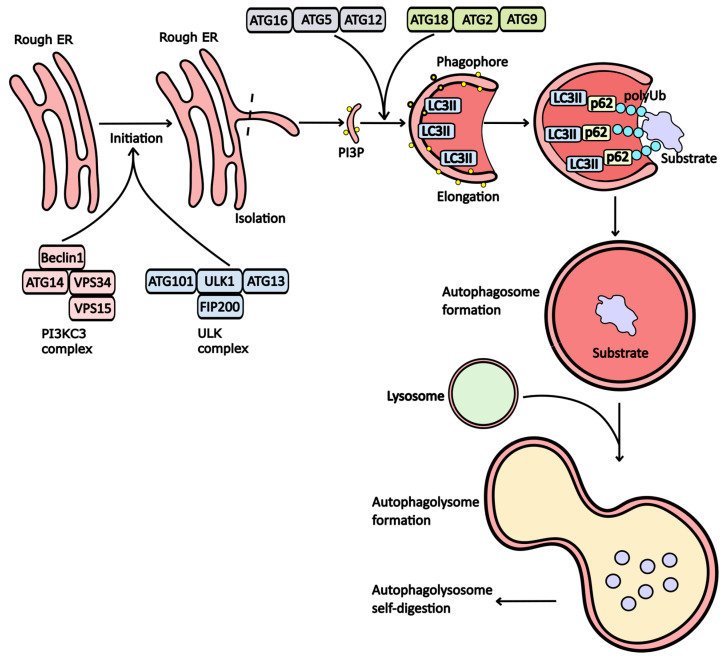
The stages of autophagy. The initiation of autophagy begins with the assembly of the PI3KC3 and ULK1 complexes. Under normal conditions, proteins within these complexes remain inactive due to mTORC1 activity. However, when ATP and nutrient levels decline, this leads to the assembly of PI3KC3 and ULK1. These assembled complexes promote the protrusion of the endoplasmic reticulum (ER) membrane, enriching it with phosphoinositol triphosphate (PI3P). For further maturation of the phagophore, the conjugating complex (Atg16/Atg5/Atg12) modifies the LC3 receptor, facilitating its integration into the membrane and bending it to form the phagophore. The Atg18/Atg2/Atg9 complex further enriches the phagophore membrane with PI3P. LC3II plays a crucial role in attaching substrates to the phagophore membrane via p62 adapter proteins. Shortly after substrate incorporation, a vesicle known as an autophagosome forms, which subsequently fuses with a lysosome to create an autophagolysosome, where substrate degradation occurs.

Currently, several forms of autophagy have been identified, including macroautophagy, microautophagy, chaperone-mediated autophagy (CMA), and crinophagy. Although these processes are morphologically distinct, they all ultimately lead to the delivery of cellular cargo to the lysosome for degradation and recycling. The most prevalent form is macroautophagy, a vesicular process crucial for the degradation of dysfunctional organelles and misfolded or aggregated proteins. This pathway plays a vital role in maintaining cellular homeostasis. During macroautophagy, the cell generates a double-membrane structure known as the phagophore, which subsequently matures into an autophagosome. The formation of an autophagosome around a lysate substrate is a central event in the autophagy process. This process involves the formation of a membrane around the substrate, which is a multistep process involving many signaling pathways and protein complexes. The first stage of autophagy is its initiation. During this stage, autophagy initiation complexes are assembled. These complexes are involved in the formation of the precursor to the autophagosomal membrane, the phagophore. The autophagic activity begins with the assembly of ULK (Unc-51-like autophagy-activating kinases) complexes consisting of FIP200 (focal adhesion kinase family interacting protein of 200 kD), Atg13 (autophagy-related protein 13), and ULK1/ULK2 proteins (Figure 1). Normally, mTORC1 complex—mTOR (mechanistic target of rapamycin) complex 1, which acts as the main negative regulator of their assembly, inhibits Atg13 by binding to its protein. In a pathological state of cells associated with a lack of ATP, mTOR activity decreases, leading to the loss of its inhibitory effects on the ULK complex. The assembled ULK then initiates the formation of another autophagic complex called PI3KC3 (another term for the VPS34 complex). This complex consists of Beclin1, Atg14/UVRAG (autophagy-related protein 13/UV radiation resistance-associated gene protein), VPS15 (vacuolar protein sorting-associated protein 15), and VPS34 proteins [1,2,3]. The next stage of autophagy is isolation. During this process, the autophagic initiator complexes formed during the initiation of autophagosome formation bind to the membrane of the endoplasmic reticulum (ER). This causes the protrusion of the ER membrane region and saturation of this region with phosphatidylinositol 3-phosphate (PI3P) molecules, which are realized with the participation of phosphoinositide 3-kinase C3 (PI3KC3) proteins [4].

The protruding portion of the membrane fully detached from the ER and transformed into a phagophore. This process involves the LC3 protein (microtubule-associated protein 1A/1B-light chain 3), which conjugates with phosphatidylethanolamine (PE) and binds to the inner surface of the membrane, forming an autophagosome. At this stage, the modified form of LC3 is referred to as LC3II. The primary complex responsible for LC3 conjugation and the curvature of phagophore membranes consists of Atg16, Atg5, and Atg12 [5].

Additionally, the formation of an autophagosome requires the enrichment of membranes with phosphatidylinositol 3-phosphate (PI3P) molecules. This enrichment is facilitated by the Atg18, Atg2, and Atg9 protein complex, which transports PI3P from the ER membrane [6]. Once these modifications are complete, substrates can associate with the phagophore membrane. However, substrates cannot attach directly to the phagophore; they require adapter proteins such as p62 and/or NBR1 (Neighbor of BRCA1 gene 1 protein). These proteins mediate the binding of substrates to the LC3II receptors (Figure 1).

Subsequently, the phagophore evolves into a double-membrane vesicle known as an autophagosome. For effective substrate degradation during autophagy, proteolytic enzymes must be present within this vesicle. To achieve this, the autophagic vesicle must fuse with a lysosome, resulting in the formation of an autolysosome. Within this structure, lysosomal enzymes break down the initial substrate.

Autophagy is a crucial mechanism for cell survival, playing a significant role in the management of pathological changes linked to various diseases. This has spurred the development and testing of drugs that can modulate autophagy, either as inhibitors or inducers. However, it is important to note that many currently synthesized drugs lack specificity for particular targets. Consequently, their effects on autophagy can be complex and context-dependent, influenced by factors such as the experimental model, cell line, and methods employed to assess drug activity. This variability has implications for clinical practice and the outcomes of clinical trials involving these compounds. This review focuses on drugs that are currently used or hold promise for clinical application, particularly in relation to specific diseases. We will explore both inhibitory and activating effects on autophagy, highlighting their potential therapeutic relevance.

## 2. Drugs That Modulate Autophagy Activity

Currently, a huge number of drugs have been developed to modulate autophagy, influencing this crucial cellular process either directly or indirectly. These compounds can be broadly categorized into those that inhibit autophagy and those that activate it. Below, we outline some of the primary groups of drugs frequently employed in preclinical and clinical studies.

### 2.1. Inhibitors of Autophagy

Autophagy inhibitors can be categorized based on their mechanisms of action into several groups: phosphoinositide 3-kinase (PI3K) inhibitors, Vps34 kinase inhibitors, vATPase (proton pump) inhibitors, ULK1 protein inhibitors, and agents that disrupt lysosomal function.

#### 2.1.1. PI3K Inhibitors

The PI3K/Akt/mTOR signaling pathway plays a crucial role in regulating cell survival. Consequently, drugs targeting the proteins involved in this pathway significantly influence autophagy activity. PI3K is classified into three main classes, each with distinct cellular functions:

- Class 1 PI3K (PI3KC1): Involved in the AKT signaling cascade and insulin receptor signaling.
- Class 2 PI3K (PI3KC2): Plays a role in cell adhesion.
- Class 3 PI3K (PI3KC3): Central to the formation of autophagosomes.

Most known PI3K inhibitors affect various classes of PI3K, leading to diverse impacts on autophagy regulation. Notably, many of these inhibitors also target proteins unrelated to PI3K signaling, which can indirectly influence autophagy. Inhibitors that selectively target PI3KC3 tend to have the most significant effect on autophagy inhibition.

**Wortmannin** is a well-known inhibitor that is used extensively in preclinical studies. Derived from the fungus Penicillium funiculosum, it features a steroid structure and inhibits all three classes of PI3K. By disrupting the PI3K/Akt/mTOR signaling pathway, Wortmannin induces cell death. Specifically, its inhibition of PI3KC3 hampers the enrichment of the phagophore membrane with PI3P molecules, thereby reducing the efficiency of phagophore formation [7].

**3-Methyladenine (3-MA)** is another widely utilized autophagy inhibitor that targets both PI3KC1 and PI3KC3. While its inhibition of PI3KC1 is irreversible, its effect on PI3KC3 is temporary, allowing for reversible autophagy inhibition [8]. 3-MA has been used in studies investigating the molecular underpinnings of atherosclerosis, coronary heart disease, and various cancers. A clinical trial has also assessed its efficacy in treating mantle cell lymphoma [9].

**LY294002** is a specific inhibitor of PI3KC1 that effectively inhibits the PI3K/Akt/mTOR pathway and enhances the expression of LC3, a marker associated with autophagy.

Its role in regulating autophagy is quite contradictory; it can act as either an autophagy inhibitor or an autophagy inducer, depending on the cell lines used and the methods employed in the study [10,11,12]. It was shown that both inhibitors, Wortmannin and LY294002, inhibit autophagy in isolated rat hepatocytes, reducing the efficiency of autophagosome formation [10]. On the contrary, treatment of gastric cancer cells (SGC7901) with LY294002 has been shown to induce autophagy and apoptosis linked to the activation of p53, caspase-3, and PUMA (p53 upregulated modulator of apoptosis). Activation of p53 [11]. A modified version, SF1126, has been explored in clinical trials for neuroblastoma treatment. SF1126 is a conjugate of LY294002 and the RGDS tetrapeptide (H-Arg-Gly-Asp-Ser-OH), which facilitates effective interaction with neuroblastoma cells enriched with integrins. This tetrapeptide is known for its high affinity for integrins due to its structural similarity to fibronectin. Unfortunately, the Phase 1 clinical trial for SF1126 was halted due to an insufficient number of participants.

Other notable PI3K inhibitors include **ZSTK474** (a PI3KC1 inhibitor), **GSK-2126458** (which targets both PI3KC1 and PI3KC3), and **PT210** (also a dual inhibitor of PI3KC1 and PI3KC3). Based on the established targets and general mechanisms of action of PI3K inhibitors known to influence autophagy, it can be inferred that these inhibitors may also impact autophagy. However, it is important to note that the nature of this effect is likely to be dual-faceted.

#### 2.1.2. Inhibitors of the PI3KC3 Complex (Vps34)

**Figure 2:**
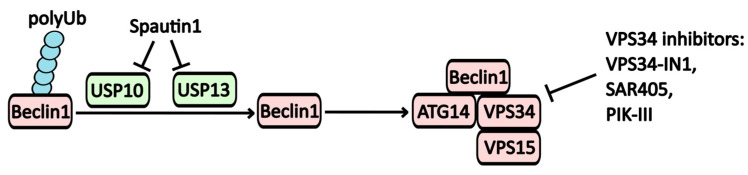
The regulatory mechanisms affecting the Vps34 complex. One method for modulating Beclin-1 activity involves its polyubiquitilation. Beclin-1, when polyubiquitinated at Lys11, inhibits the assembly of the PI3KC3 complex. Spautin-1 inhibits the USP10 and USP13 ubiquitin-specific peptidase activity, thereby disrupting the assembly of the PI3KC3 complex, which includes Beclin-1, ATG14, VPS34, and VPS15.

Inhibition of the Vps34 complex disrupts autophagy at its early stages. Several direct inhibitors of Vps34 have been identified, including **VPS34-IN-1**, **Compound-31**, **SAR405,** and **PIK-III** (Figure 2). Notably, Spautin-1 warrants special attention due to its unique mechanism of action. It directly inhibits ubiquitin-specific proteases USP10 (ubiquitin-specific peptidase 10) and USP13 (ubiquitin-specific peptidase 13), which are responsible for degrading polyubiquitin at the Lys-11 position of the Beclin-1 protein. This degradation inactivates Beclin-1, a crucial component for the formation of the PI3KC3 complex and the assembly of autophagic vesicles [13,14]. Consequently, Spautin-1 may significantly impact protein degradation mechanisms. In studies involving neuroblastoma cells, treatment with Spautin-1 has been shown to decrease p53 levels due to enhanced proteolysis and impaired deubiquitination [14].

Furthermore, Spautin-1 inhibits the EGFR signaling pathway, which contributes to a reduced survival rate of cancer cells [13], positioning this molecule as a promising candidate for potential cancer therapies. However, it is important to note that no clinical trials involving Spautin-1 have been conducted to date.

#### 2.1.3. ULK1 Inhibitors

The ULK1 complex plays a crucial role as the primary initiator of autophagy. Disruption of its assembly impairs the initiation of this essential cellular process.

**MRT68921** is a notable inhibitor of ULK1, and its application in acute myeloid leukemia cells has been linked to a decreased survival rate. While MRT68921 inhibits ULK1, it may also lead to the accumulation of LC3II and a reduction in phosphorylated levels of Atg13 and ULK (Ser555), which could theoretically induce autophagy. However, these effects are overshadowed by the predominant suppression of ULK activity, and the primary outcome of MRT68921 is the inhibition of autophagic processes. Additionally, MRT68921 suppresses the phosphorylation of eIF2 and PERK, resulting in reduced endoplasmic reticulum (ER) stress and subsequent inhibition of autophagosome formation [15]. Notably, MRT68921 has been shown to arrest the cell cycle in the G2 phase when applied to chronic lymphocytic leukemia cells. The combination of MRT68921 with the Bcl-2 inhibitor Venetoclax has demonstrated a synergistic effect in suppressing cell proliferation [16]. Other ULK1 inhibitors include Compound 6 and SBI-0206965.

#### 2.1.4. V-ATPase (Proton Pump) and Lysosomal Inhibitors

**Figure 3:**
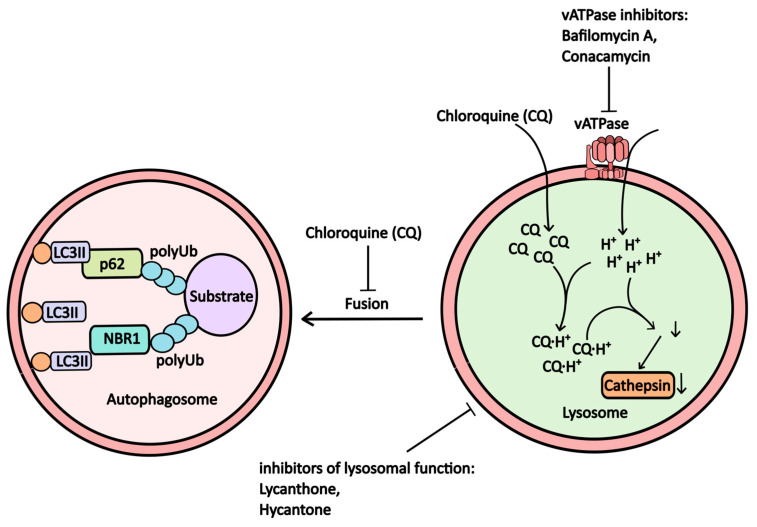
The scheme illustrates how proton pump inhibitors, which alter lysosomal pH and microsomal function, affect autophagy (further explanations are provided in the accompanying text).

Inhibition of proton pump activity on the lysosomal membrane disrupts lysosomal function due to decreased activity of lysosomal proteolytic enzymes, which require an acidic pH for optimal function. Concanamycin and Bafilomycin A1 are well-known inhibitors that interfere with proton pump operation (Figure 3).

**Bafilomycin A1**, an antibiotic derived from *Streptomyces griseus*, inhibits V-ATPase in lysosomes, leading to increased lysosomal pH and suppressed autophagy (Рисунoк 3). It also stabilizes the Beclin-1/Bcl-2 heterodimer, resulting in reduced active Beclin-1 levels and further inhibition of autophagic processes [17,18]. While bafilomycin A1 is frequently used in in vitro studies to model lysosomal dysfunction, its clinical application has been limited due to toxicity concerns.

For effective autophagy, fusion between autophagosomes and lysosomes is essential, forming autophagolysosomes where substrate degradation occurs. In cases of lysosomal dysfunction, autophagosomes may fail to form or may do so incompletely, impairing the activity of proteolytic enzymes such as cathepsins.

**Chloroquine** and its derivative, hydroxychloroquine, are among the most commonly used inhibitors of autophagy in clinical trials. Both alkaloids are derivatives of 4-aminoquinolines, with chloroquine having a long-established role in malaria treatment. The drug has garnered renewed interest for potential repurposing due to its nitrogen-rich heterocyclic aromatic structure, which undergoes protonation within lysosomes. This process raises lysosomal pH, disrupting the function of hydrolytic enzymes (Figure 3) [18] and diminishing substrate degradation efficiency within autophagosomes, as well as impairing the fusion between lysosomes and autophagosomes [19].

Currently, chloroquine is utilized as an immunosuppressive agent for conditions such as rheumatoid arthritis and systemic lupus erythematosus. Its antiviral efficacy has been demonstrated in several studies [20]. Furthermore, the efficiency of chloroquine against SARS-CoV-2 was demonstrated, but the results from phase 4 clinical trials (NCT04331600) have raised concerns about its effectiveness for COVID-19 treatment. Ongoing research continues to explore the potential of chloroquine in cancer therapy (Table 1). Other compounds exhibiting similar cellular effects include Lys05 and ARN 5187.

Another drug that directly impacts lysosomal function is **lucanthone**. This compound increases lysosomal membrane permeability, leading to the release of cathepsin D and subsequent lysosomal dysfunction. This dysfunction results in reduced efficiency of autophagosome formation and the induction of apoptosis [21]. Notably, while lucanthone treatment promotes the binding of the LC3 protein to autophagosomes [21], this effect is minimal compared to the overarching lysosomal impairment.

In addition to its effects on autophagy, lucanthone inhibits DNA repair mechanisms and disrupts DNA replication, ultimately causing cell cycle arrest and apoptosis through the inhibition of apurinic endopeptidase 1 (APE1) [22].

The efficacy of lucanthone, in combination with the antitumor alkylating agent temozolomide (TMZ), was evaluated in a clinical trial (NCT01587144) for the treatment of glioblastoma. Another study (NCT0201454) assessed its potential to enhance the effectiveness of radiotherapy for non-small cell lung cancer metastases in the brain.

Currently, lucanthone is used as an antihelmintic, with its effects linked to the inactivation of serotonin 5-HT receptors, resulting in tetany (uncontrolled muscle contractions) in nematodes such as *S. haematobium* and *S. mansoni*, ultimately leading to their death. Its analog, Hycanthone, exhibits a similar mechanism of action on eukaryotic cells. 

Cathepsins are crucial for lysosomal proteolytic activity; thus, inhibitors of cathepsins also function as autophagy inhibitors [23]. Notable cysteine protease inhibitors include **E64d (Aloxistatin)**, **E64c**, **Leupeptin**, **k777**, and **Odanacatib**. These compounds possess a broad range of effects and can inhibit various proteases, including those not directly associated with lysosomal activity.

### 2.2. Inducers of Autophagy

Drugs that activate autophagy can be categorized based on their mechanisms of action: mTOR inhibitors, ER stress inducers, IMRase (inositol monophosphatase) inhibitors, MTMR14 (myotubularin-related protein 14)/jumpy inhibitors, mTOR-independent autophagy inducers, calpain inhibitors, calcium channel blockers, Bcl-2 inhibitors, AMPK (5′ AMP-activated protein kinase) activators, and TFEB (Transcription factor EB) activators.

#### 2.2.1. mTOR Inhibitors

The mTOR protein is a key component of the mTORC1 complex, which negatively regulates the activity of TFEB and ULK complexes, thereby inhibiting autophagy. Drugs that suppress mTOR (mechanistic target of rapamycin), such as Rapamycin and Everolimus (RAD001) and Temsirolimus (AP23576), stimulate autophagy by inhibiting this pathway.

**Rapamycin**, an antibiotic derived from *Streptomyces hygroscopicus*, has immunosuppressive properties and is often used to prevent organ rejection in transplantation. By inhibiting mTOR within the mTORC complex, rapamycin activates Atg13 and initiates autophagy (see Figure 1) [24,25].

Beyond its role in autophagy, mTOR also regulates translation. It is part of a complex known as the mTOR/Raptor/GβL complex, which inhibits the activity of 4E-BP1—a negative regulator of eIF4—and activates S6K, a protein involved in ribosome assembly. Consequently, rapamycin can also inhibit translation by blocking this complex’s activity.

Rapamycin has been extensively studied in over 700 clinical trials to explore its potential for treating various conditions. The research includes investigations into its combination with trastuzumab for HER2+ breast cancer (Phase 2, NCT01827943) and its efficacy in enhancing radiotherapy outcomes for rectal cancer (Phase 2, NCT00409994).

**Temsirolimus**, a prodrug of rapamycin, has also been evaluated in phase 2 trials for bladder (NCT01827943) and prostate (NCT00919035) cancers.

**Everolimus**, like Temsirolimus, is a modified rapamycin derivative used to mitigate rejection reactions in kidney and liver transplants while also exhibiting anti-cancer properties. The potential of everolimus in treating malignant diseases has been explored in several clinical trials, including its efficacy in combination with prednisone for kidney cancer (NCT02479490).

#### 2.2.2. ER-Stress Inductors

The induction of ER stress leads to increased expression of genes encoding autophagy proteins, particularly through the PERK/eIF2/ATF4 (Protein Kinase RNA-Like ER Kinase Eukaryotic Initiation Factor 2/Activating transcription factor 4) pathway. Key inducers of ER stress include Tunicamycin, Thapsigargin, and Brefeldin A. Disruption of the ubiquitin-proteasome system is a major factor associated with the development of ER stress. Proteasome inhibitors such as Bortezomib and NPI-0052 act as indirect activators of autophagy by inhibiting proteasomal activity.

**Brefeldin A**, an antibiotic derived from the fungus Eupenicillium brefeldianum, disrupts vesicular transport between the endoplasmic reticulum and the Golgi apparatus. It inhibits GBF1 (Golgi-specific brefeldin A-resistance guanine nucleotide exchange factor 1), which normally exchanges GDP for GTP, thereby regulating Arf1 (ADP-ribosylation factor 1) activity involved in COPI (Coat protein I) vesicle formation on the Golgi membrane. This disruption leads to impaired homeostatic regulation of ER protein transport [26], resulting in ER stress.

Notably, brefeldin A has been shown to activate gene expression and proteins involved in autophagy. Treatment of liver cancer cells with brefeldin A significantly increases the accumulation of LC3II, ATG5, and Beclin1 proteins in the cytoplasm, effectively activating autophagy [27].

**Bortezomib**, marketed under the trade name Velcade, targets the 20S core of the 26S proteasome, disrupting its function and inducing ER stress and autophagy [28,29]. In clinical practice, bortezomib is utilized as an anti-cancer agent for multiple myeloma treatment, with numerous clinical trials investigating its use alone or in combination with other therapies [30,31].

**NPI-0052** (Marizomib, Salinosporamide A) is another drug that inhibits the 26S proteasome. The molecular structure of this drug includes a β-lactone ring, which provides binding to threonine in the structure of β5i, β2i, and β1i subunits of the 20S proteasome complex. This interaction disrupts protein degradation and induces autophagy via ER stress [31]. Currently, NPI-0052 is undergoing clinical trials for multiple myeloma treatment.

#### 2.2.3. Inhibitors of Inositol Monophosphatase (IMPase)

Inositol diphosphatase plays a crucial role in hydrolyzing phosphodiester bonds in inositol diphosphate, converting it into inositol monophosphate. This phosphodiesterase activity is essential for maintaining cellular inositol levels; disruptions can lead to reduced triphosphorylated inositol and subsequent autophagy induction. Lithium salts, such as lithium carbonate and lithium chloride, along with their specific inhibitor L-690/330, are commonly used as IMRase inhibitors. Additionally, drugs that interact directly with inositol, like carbamazepine and valproic acid, can interfere with IP3 accumulation [32].

**L-690/330** acts as a competitive inhibitor of IMRase (inositol monophosphatase) [33]. Treatment with this drug has been shown to induce autophagy in cells. Disruption of inositol triphosphate (IP3) turnover reduces the activity of the IP3R Ca^2+^ channel, leading to decreased cytoplasmic Ca^2+^ levels [34]. Calcium plays a dual role in regulating autophagy—both activating and inhibiting it—but L-690/330 specifically enhances autophagic activity.

#### 2.2.4. MTMR14 (Jumpy) Inhibitors

**Figure 4:**
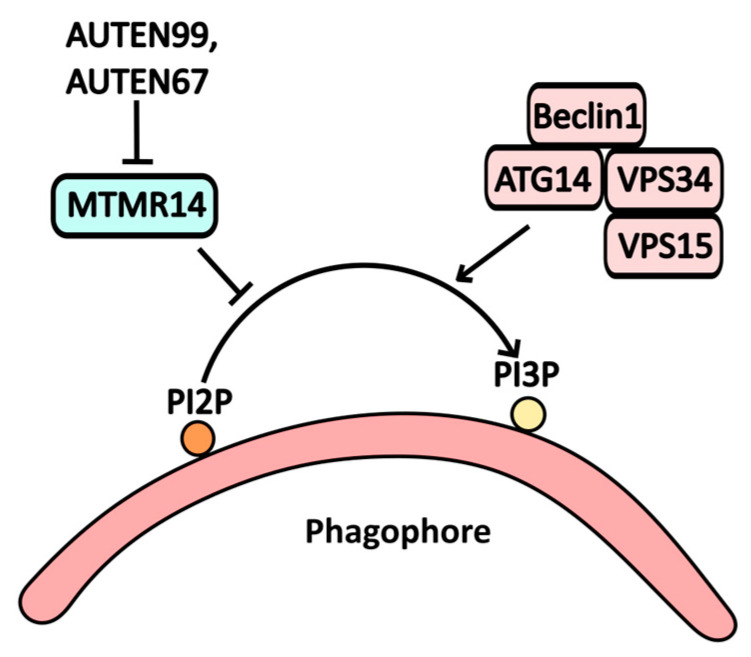
The scheme illustrates the action of AUTEN99 and AUTEN67 on autophagy, where the MTMR1/Jumpy protein acts as a functional antagonist to the PI3K complex. This interaction leads to the degradation of phosphoinositol phosphate PI3P at the phagosome membrane, disrupting autophagy. By inhibiting MTMR activity, AUTEN99 and AUTEN67 facilitate the accumulation of PI3P at the phagosome membrane.

MTMR14, a myotubularin-binding protein, serves as an inhibitor of autophagy due to its phosphoinositide phosphatase activity. It hydrolyzes PI3P on the autophagic membrane to PI2P, thereby suppressing autophagic assembly. Consequently, the inhibition of MTMR14 activates autophagy [35]. Compounds such as **AUTEN99** and **AUTEN67** have been identified as potential inhibitors of MTMR14 (Figure 4).

As a relatively new synthetic molecule, **AUTEN99** blocks the phosphatase activity of MTMR14, preventing the conversion of PI3P to PI2P [35]. While it is not yet employed in clinical practice, experimental studies have shown that AUTEN99 reduces α-synuclein protein aggregates in the nervous tissue of Drosophila models of Parkinson’s disease [36].

#### 2.2.5. mTOR-Independent Inducers

This category includes drugs that induce autophagy without directly interacting with components of the mTORc1 complex. Instead, they activate signaling pathways that influence mTORc1 activity. Notable compounds in this group include **Corynoxine** and **W09**.

**Corynoxine**, an indole alkaloid derived from the *Uncaria* plant in the Rubiaceae family, has been shown to activate autophagy. This effect is linked to the induction of the PI3KC3 complex and a reduction in the phosphorylation levels of mTOR, AKT, and p70S6K [37]. Although not currently utilized in conventional medicine, Corynoxine has been employed in traditional Chinese medicine for its hypotensive properties and has demonstrated potential in reducing α-synuclein and β-amyloid levels in neurons [38], making it a promising candidate for treating neurodegenerative diseases.

**W09** functions as an activator of autophagy and apoptosis dependent on Atg7. Treatment with W09 enhances the transcription of the p62 gene and stimulates autophagy via the EGFR/RAS/RAF/MAP2K/MAPK1-3 pathway. This mechanism was confirmed in studies using the MAPK 1-3 inhibitor U0126, which inhibited MAPK (Mitogen-Activated Protein Kinase) activity and subsequently reduced both autophagic and caspase-dependent apoptotic responses induced by W09. Additionally, W09 can activate PARP, a protein involved in DNA repair and autophagy via the AMP/LKB (liver kinase B)/AMPK signaling pathway [39]. Despite promising experimental data, clinical studies involving this molecule have yet to be conducted.

#### 2.2.6. Calpain Inhibitors

**Figure 5:**
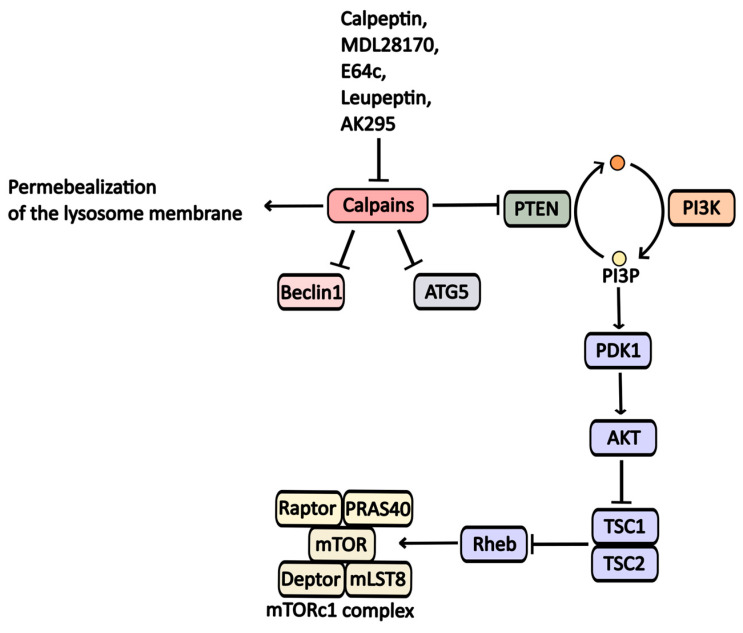
Calpaine inhibitors and their impact on autophagy regulation. Calpaines suppress Beclin1, ATG5, and PTEN, ultimately inhibiting autophagic processes.

Calpains, a family of calcium-dependent cytoplasmic proteases, play a significant role in regulating various signaling cascades associated with cell survival. One mechanism by which calpains inhibit autophagy is through the degradation of PTEN (phosphatase and tensin homolog) phosphatase, leading to PI3P accumulation and inhibition of the TSC1/TSC2 (tuberous sclerosis 1/2) complex. This sequence results in increased GTP-bound Rheb levels, which activate the mTORC1 complex and subsequently inhibit autophagic activity [40] (Figure 5).

Furthermore, calpains contribute to the degradation of key proteins involved in phagophore formation, such as Atg5 and Beclin1 [41].

Calpains also affect lysosomal function; they can permeabilize lysosomal membranes, causing dysfunction. Inhibiting calpain activity has been shown to enhance autophagy.

Protease inhibitors like **Calpeptine**, **MDL-28170**, **E64c**, **AK295,** and **Leupeptine** target calpain enzymes and exhibit a broad range of activity against them [42].

**MDL-28170** is a calpain inhibitor. It was found that this molecule can penetrate the hemato-encephalic barrier and inhibit the work of calpains. It was found that the course of therapy of this drug in laboratory mice with a model of brain injury (FPE—fluid percussion injury) leads to a pronounced neuroprotective effect and has a stimulating effect on damage healing [43].

**AK-295**. Like MDL28170, it causes neuroprotective effects and is a calpain inhibitor. In mice with damaged spinal cord, AK295 reduces calpain-induced apoptosis and improves the functioning of neurons [44].

**Leupeptin**. It is an inhibitor of calpain 5 and cathepsins B6, H, and L7 [45]. Calpeptin is an inhibitor of calpain and caspase-3. Using a model of cerebral ischemia in mice, it was found that Calpeptin reduces the induction of apoptosis of nerve cells in the CA1 region of the hippocampus [46].

#### 2.2.7. Bcl-2 Inhibitors

**Figure 6:**
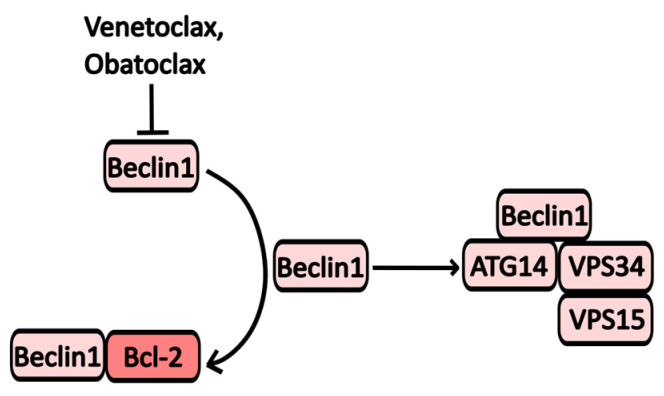
The scheme of Obatoclax and Venetoclax action on autophagy. Inhibition of Bcl-2 leads to an accumulation of Beclin-1, which forms complexes that promote the formation of autophagosomes.

One mechanism by which Bcl-2 influences autophagy involves the formation of heterodimeric complexes with key autophagy proteins, such as Bcl-2/Beclin-1 and Bcl-2/BNIP3 (BCL2/adenovirus E1B 19 kDa protein-interacting protein 3). Notable inhibitors of Bcl-2 include the broad-spectrum inhibitor **Obatoclax** and the selective Bcl-2 inhibitor **Venetoclax** (Figure 6). Venetoclax is primarily used in the treatment of chronic lymphocytic leukemia (CLL), and its mechanism of action on autophagic activity is linked to its ability to activate Beclin-1 by releasing it from its complex with Bcl-2 [16].

**Obatoclax** (GX15-070) targets various members of the Bcl-2 family, including Bcl-XL, Mcl-1, and Bcl-2 [47]. By inhibiting these proteins, Obatoclax triggers mitochondrial apoptosis and the formation of oligomeric channels in the mitochondrial outer membrane, exerting effects on autophagy similar to those of Venetoclax. Additionally, treatment with Obatoclax activates Atg7 and Atg5, enhancing LC3 processing and initiating PI3KC3 assembly (see Figure 1) [47,48,49,50].

However, some studies indicate that Obatoclax may also suppress autophagy due to decreased lysosomal activity resulting from cathepsin inhibition [51]. It has been shown to induce cell death in Hodgkin’s lymphoma, acute myeloid leukemia, and small-cell lung cancer cells. Currently, several clinical trials are underway to evaluate the efficacy of Obatoclax in combination with other anti-cancer agents. For instance, combinations with rituximab, a monoclonal CD20 inhibitor for follicular lymphoma (NCT00427856), and bortezomib for multiple myeloma (NCT0719901) are being investigated. As a standalone treatment, Obatoclax has shown promise for acute myeloid leukemia (NCT68491), with over 30 trials conducted to date.

#### 2.2.8. AMPK Activators

**Figure 7:**
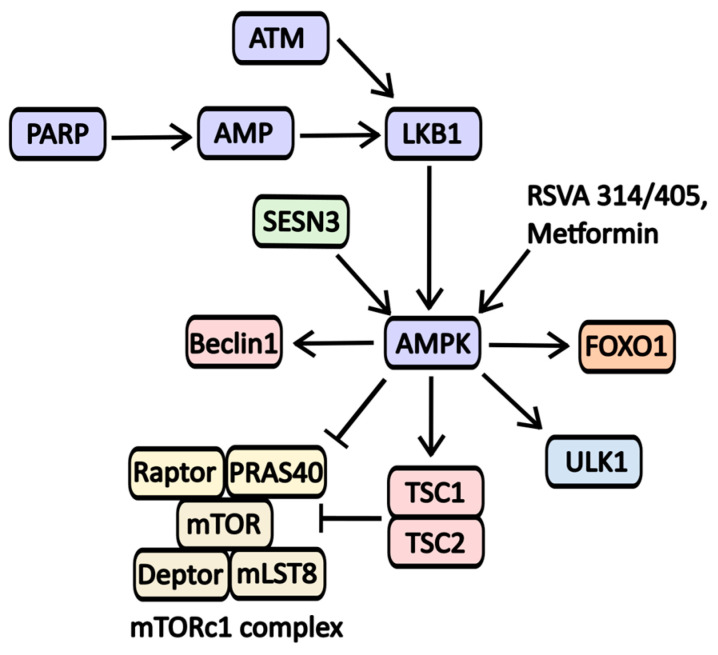
The scheme of AMPK activators action on signaling pathways involved in autophagy regulation. One of the key triggers for AMPK activation is the accumulation of AMP, often resulting from nutrient deprivation or cellular stress from various sources, including DNA damage. The DNA repair protein PARP has been shown to activate AMPK; however, this process requires NAD·H and ATP. A decreased level of ATP levels leads to activation of LKB1, resulting in AMPK phosphorylation and activation. LKB1 also relies on ATM-kinase, another protein involved in DNA repair. Furthermore, AMPK activity is influenced by metabolic regulators such as Sestrin-3 (SESN3). AMPK promotes autophagy by inhibiting mTORC1 activity while activating essential proteins like Beclin-1, ULK1, and the mTORC inhibitor complex TSC-1/2.

AMPK serves as a crucial nutrient sensor, activating in response to significant AMP (adenosine monophosphate) accumulation, which occurs during starvation or catabolic states. AMP activators include **RSVA 314/405** and Metformin (Figure 7) [52].

**Metformin** is widely recognized for its ability to activate AMP-activated protein kinase (AMPK), primarily through the inhibition of the first complex in the electron transport chain. This activation occurs in response to metabolic stress and is linked to the regulation of numerous signaling molecules involved in autophagy (see Figure 7). While Metformin is primarily used to manage type 2 diabetes mellitus, it is also being explored as a potential treatment for various other conditions. Notably, its effectiveness has been evaluated in combination with chloroquine for cancers with IDH1/2 (Isocitrate dehydrogenase 1 and 2) mutations. Additionally, Metformin’s impact has been studied in the context of lung adenocarcinoma (Phase 2 trials), vitiligo (NCT05607316), polycystic ovary syndrome (Stein-Leventhal syndrome, NCT03086005), childhood obesity (NCT02274948), and glaucoma (NCT05426044).

#### 2.2.9. TFEB Activators

TFEB is a transcription factor that regulates the expression of genes containing the so-called CLEAR (Coordinated Lysosomal Expression and Regulation) element in their sequence. Many of these genes encode proteins involved in the initiation and formation of autophagosomes and in the biogenesis of lysosomes and endosomes, in particular, p62, UVRAG, LC3, ATG5, and Beclin-1. In the case of cellular stress, there is a decrease in the amount of the phosphorylated form of TFEB as a result of a decrease in the activity of mTORC1. This leads to its release from the 14-3-3σ/TFEB complex and promotes its transport to the nucleus, where its transcriptional activity is carried out. MSL-7 can be considered a drug that causes dephosphorylation of TFEB and its activation. The mechanism of action of this drug is associated with the activation of calcineurin, a cellular phosphatase involved in the phosphorylation of TFEB [53]. It has been shown in laboratory animal models (mice) that MSL-7 reduces the accumulation of hIAPP amyloid (human islet amyloid polypeptide) in the islets of pancreatic Langerhans and prevents the development of a local inflammatory process, as well as associated damage to pancreatic tissues [54]. MSL-7 has been found to reduce glucose tolerance in pancreatic β-cells and promote the survival of insulin-producing cells [54]. In this regard, this drug may be promising for the treatment of diabetes mellitus, but clinical trials have not yet been conducted.

## 3. Conclusions

Macroautophagy is a crucial mechanism for normal cell functioning. It plays a vital role in cell survival, as it directly or indirectly affects the regulation of autophagic processes. Disruption of these mechanisms has been linked to the development and progression of various diseases, making them promising targets for treatment. Many inhibitors and activators of autophagy have been tested in clinical trials, either in combination with other medications or as monotherapy agents (Table 2). Despite promising initial results, there are still many aspects of their mechanisms that remain unknown, making them of significant interest to scientists.

## Figures and Tables

**Table 1:** Clinical trials of chloroquine.

Disease	NCT Number	Phase of Clinical Trial	Drugs That Were Used in Combination	Subtypes of Disease
Small cell lung cancer	NCT01575782	I-III (Canceled)	-----//-----	-----//-----
Breast cancer	NCT02333890	II	-----//-----	Breast cancer, Invasive breast cancer
Metastatic breast cancer	NCT01446016	II (Completed)	(a) Chloroquine + Paclitaxel (b) Chloroquine + Docetaxel (c) Chloroquine + Abraxane (d) Chloroquine + Ixabepilon	Breast neoplasia, Breast cancer
Stage 4 small cell lung cancer	NCT00969306	I (Canceled)	-----//-----	-----//-----
Dense tumors	NCT02071537	I (Completed)	(a) Chloroquine + Carboplatin (b) Chloroquine + Gemcitabine	-----//-----
Pancreatic cancer	NCT01777477	I (Completed)	Chloroquine + Gemcitabine	----//-----
Intraductal carcinoma	NCT01023477	I-II (Completed)	-----//-----	Intraductal non-filtering carcinoma, Ductal carcinoma
Dense tumors with IDH1/2 mutation	NCT02496741	I-II	Chloroquine + Metformin	Glioma, Cholangiocarcinoma, Chondrosarcoma
Brain metastases from dense tumors	NCT01894633	II (Canceled)	Chloroquine + radiotherapy	-----//-----
Brain metastases with IDO2 genetic status	NCT01727531	(Canceled)	Chloroquine + radiotherapy	-----//-----
Brain cancer	NCT04397679	Recruitment for clinical trials	Chloroquine, intensity modulated radiation therapy (IMRT), Temolozomide, tumor treating field therapy (TTF)	Glioblastoma, Gliosarcoma
Recurrent and refractory multiple myeloma	NCT01438177	II (Completed)	Chloroquine + Bortezomib + Cyclophosphamide	Multiple myelomas
Melanoma	NCT01469455	I	DT01 + Chloroquine + radiotherapy	-----//-----
Glioblastoma	NCT00224978	III (Completed)	-----//-----	Glioblastoma multiform
Glioblastoma	NCT02432417	II (Recruitment for clinical trials)	Chloroquine + Temolozomide + radiotherapy	-----//-----

**Table 2:** Summarizing the table of drugs discussed in the review.

Drug	Group	Targets	Application in Medicine	Clinical Trial
**Wortmannin**	PI3K inhibitor	PI3KC1, PI3KC2, PI3KC3	-----//-----	-----//-----
**3-Methyladenine** **(3-MA)**	PI3K inhibitor	PI3KC1, PI3KC3	-----//-----	-----//-----
**LY294002**	PI3K inhibitor	PI3KC1, p53, LC3, Caspase-3, PUMA	-----//-----	**NCT02337309** (SF1126 for Patients with Neuroblastoma)
**ZSTK474**	PI3K inhibitor	PI3KC1	-----//-----	**NCT01682473** (A Study of ZSTK474 in Japanese Patients with Advanced Solid Malignancies)
**GSK-2126458**	PI3K inhibitor	PI3KC1, PI3KC3	-----//-----	**NCT00972686** (Dose-Escalation Study of GSK2126458), **NCT01725139** (A Proof of Mechanism Study with GSK2126458 in Patients with Idiopathic Pulmonary Fibrosis), **NCT01248858** (Study to Investigate the Safety, Pharmacokinetics, Pharmacodynamics, and Clinical Activity of GSK2126458 and GSK1120212 Combination Therapy in Subjects with Advanced Solid Tumors).
**PT210**	PI3K inhibitor	PI3KC1, PI3KC3	-----//-----	-----//-----
**Spautin-1**	PI3KC3-C1 (Vps34) inhibitor	USP10, USP13	-----//-----	-----//-----
**VPS34-IN-1**	PI3KC3-C1 (Vps34) inhibitor	Vps34	-----//-----	-----//-----
**Compound-31**	PI3KC3-C1 (Vps34) inhibitor	Vps34	-----//-----	-----//-----
**SAR405**	PI3KC3-C1 (Vps34) inhibitor	Vps34	-----//-----	-----//-----
**PIK-III**	PI3KC3-C1 (Vps34) inhibitor	Vps34	-----//-----	-----//-----
**MRT68921**	ULK1 inhibitor	ULK1, Atg13	-----//-----	-----//-----
**Bafilomycin A1**	vATPase inhibitor	vATPase	-----//-----	**NCT04389580** (Combination Therapy with Isotretinoin and Tamoxifen Expected to Provide Complete Protection Against Severe Acute Respiratory Syndrome Coronavirus (Combination) **NCT05711810** (Medicine-induced Cardiac Hemodialysis on COVID-19)
**Lys05**	Lysosome	Lysosome	-----//-----	-----//-----
**ARN 5187**	Lysosome	Lysosome, REV-ERBβ	-----//-----	-----//-----
**Lucanthone,** **Hycanthone**	Lysosome	Lysosomal membrane, APE1	Helmints	**NCT01587144** (Safety and Efficacy Study of Lucanthone When Used in Combination with Temozolomide (TMZ) and Radiation to Treat Glioblastoma Multiforme) **NCT02014545** (Evaluation of Lucanthone to Whole Brain Radiation Therapy in Patients with Brain Metastases From Non-Small Cell Lung Cancer)
**Rapamycin**	mTOR inhibitor	mTOR	Immunosuppressant	**NCT00411788** (Study of Rapamycin and Trastuzumab for Patients With HER-2 Receptor-Positive Metastatic Breast Cancer) **NCT00409994** (Safety Study of Rapamycin Administered Before and During Radiotherapy to Treat Rectum Cancer)
**Temsirolimus**	mTOR inhibitor	mTOR	Renal cell carcinoma	**NCT01827943** (Phase II Evaluating Efficacy of Temsirolimus in 2 Line Therapy for Patients with Advanced Bladder Cancer) **NCT00919035** (Single Agent Temsirolimus in Chemotherapy-naïve Castration-Resistant Prostate Cancer Patients)
**Everolimus**	mTOR inhibitor	mTOR	Immunosuppressant, Kidney cancer	**NCT02479490** (Prednisone Plus Everolimus in Patients with Metastatic Renal Cell Cancer After Failure of VEGFR -TKI)
**Brefeldin A**	ER-stress inductor	GBF1	-----//-----	**NCT03044509** (Diagnosis of Tuberculosis in Swiss Children)
**Bortezomib**	ER-stress inductor	26S proteasome	Multiple myeloma, mantle cell lymphoma	**NCT01328236** (Bortezomib in Combination with Liposomal Doxorubicin and Dexamethasone to Treat Plasma Cell Leukemia) **NCT00183937** (Study of Bortezomib and Docetaxel for Patients with Hormone Refractory Prostate Cancer)
**NPI-0052**	ER-stress inductor	26S proteasome	-----//-----	**NCT00461045** (Phase 2 Clinical Trial of NPI-0052 in Patients with Relapsed or Relapsed/Refractory Multiple Myeloma) **NCT00396864** (Phase 1 Clinical Trial of NPI-0052 in Patients with Advanced Solid Tumor Malignancies or Refractory Lymphoma)
**L-690/330**	IMPase inhibitors	IMPase	-----//-----	-----//-----
**lithium carbonate,** **lithium chloride**	IMPase inhibitors	IMPase	Bipolar disorder major depressive disorder	**NCT02862210** (Low-Dose Lithium for the Treatment of Behavioral Symptoms in Frontotemporal Dementia) **NCT01108068** (Trial of Lithium Carbonate for Treatment of Osteoporosis-pseudoglioma Syndrome) **NCT01096082** (Safety and Efficacy of Lithium Carbonate in Patients with Spinocerebellar Ataxia Type 3)
**Carbamazepine**	IMPase inhibitors	Inositol	Epilepsy	**NCT00203567** (Carbamazepine Extended-Release for the Treatment of Bipolar Depression) **NCT01379469** (Carbamazepine in Severe Liver Disease Due to Alpha-1 Antitrypsin Deficiency)
**Valproic acid**	IMPase inhibitors	Inositol	Epilepsy	**NCT04940572** (Efficacy Study of Daily Administration of VPA in Patients Affected by Wolfram Syndrome) **NCT03919292** (Neratinib + Valproate in Advanced Solid Tumors, w/Expansion Cohort in Ras-Mutated Ca)
**AUTEN99** **AUTEN67**	MTMR14 inhibitor	MTMR14/Jumpy	-----//-----	-----//-----
**Corynoxine**	mTOR independent inducers	PI3KC3, mTOR, AKT, p70S6K	-----//-----	-----//-----
**W09**	mTOR independent inducers	PARP, P62	-----//-----	-----//-----
**MDL-28170**	Calpains inhibitor	Calpains	-----//-----	-----//-----
**AK-295**	Calpains inhibitor	Calpains	-----//-----	-----//-----
**Leupeptin**	Calpains inhibitor	Calpain 5, cathepsins	-----//-----	-----//-----
**Obatoclax**	Bcl2 inhibitor	Bcl-XL, Mcl-1, Bcl-2	Various types of cancer	**NCT00918931** (Obatoclax for Systemic Mastocytosis) **NCT00684918** (Study of Obatoclax in Previously Untreated Acute Myeloid Leukemia)
**Venetoclax**	Bcl2 inhibitor	Bcl-2	Chronic lymphocytic leukemia	**NCT04501939** (Cirmtuzumab Consolidation for Treatment of Patients with Detectable CLL on Venetoclax) **NCT04171791** (A Study of ABT-199 (Venetoclax) for Cutaneous T Cell Lymphoma)
**Metformin**	AMPK activator	AMPK	Type 2 Diabetes	**NCT02978547** (The Effects of Neoadjuvant Metformin on Tumor Cell Proliferation and Tumor Progression in Pancreatic Ductal Adenocarcinoma) **NCT05607316** (Evaluating the Efficacy and Safety of Metformin in Vitiligo) **NCT05426044** (Metformin as a Neuroprotective Therapy for Glaucoma—A Randomized Controlled Trial)
**RSVA 314/405**	AMPK activator	AMPK	-----//-----	-----//-----
**MSL-7**	TFEB activator	TFEB	-----//-----	-----//-----

## Data Availability

The data presented in this review are available on request from the corresponding author.

## References

[1] Yu L., Chen Y., Tooze S.A. (2017). Autophagy Pathway: Cellular and Molecular Mechanisms. Autophagy.

[2] Vakifahmetoglu-Norberg H., Xia H.G., Yuan J. (2015). Pharmacologic agents targeting autophagy. J. Clin. Investig..

[3] Kocak M., Ezazi Erdi S., Jorba G., Maestro I., Farrés J., Kirkin V., Martinez A., Pless O. (2022). Targeting autophagy in disease: Established and new strategies. Autophagy.

[4] Wirth M., Joachim J., Tooze S.A. (2013). Autophagosome Formation—The Role of ULK1 and Beclin1–PI3KC3 Complexes in Setting the Stage. Semin. Cancer Biol..

[5] Lystad A.H., Carlsson S.R., Simonsen A. (2019). Toward the Function of Mammalian ATG12–ATG5-ATG16L1 Complex in Autophagy and Related Processes. Autophagy.

[6] Nagy P., Hegedűs K., Pircs K., Varga Á., Juhász G. (2014). Different Effects of Atg2 and Atg18 Mutations on Atg8a and Atg9 Trafficking During Starvation in Drosophila. FEBS Lett..

[7] Uluer T., Sonmez P.K., Akogullari D., Onal M., Tanriover G., Inan S. (2021). Do Wortmannin and Thalidomide Induce Apoptosis by Autophagy Inhibition in 4T1 Breast Cancer Cells In Vitro and In Vivo?. Am. J. Transl. Res..

[8] Wu Y.-T., Tan H.-L., Shui G., Bauvy C., Huang Q., Wenk M.R., Ong C.N., Codogno P., Shen H.-M. (2010). Dual Role of 3-Methyladenine in Modulation of Autophagy via Different Temporal Patterns of Inhibition on Class I and III Phosphoinositide 3-Kinase. J. Biol. Chem..

[9] Feng M., Wang J., Sun M., Li G., Li B., Zhang H. (2021). 3-Methyladenine but Not Antioxidants to Overcome BACH2-Mediated Bortezomib Resistance in Mantle Cell Lymphoma. Cancer Cell Int..

[10] Blommaart E.F.C., Krause U., Schellens J.P.M., Vreeling-Sindelárová H., Meijer A.J. (1997). The Phosphatidylinositol 3-Kinase Inhibitors Wortmannin and LY294002 Inhibit Autophagy in Isolated Rat Hepatocytes. Eur. J. Biochem..

[11] Xing C., Zhu B., Liu H., Yao H., Zhang L. (2008). Class I Phosphatidylinositol 3-Kinase Inhibitor LY294002 Activates Autophagy and Induces Apoptosis Through p53 Pathway in Gastric Cancer Cell Line SGC7901. Acta Biochim. Biophys. Sin..

[12] Feng Y., Gao Y., Wang D., Xu Z., Sun W., Ren P. (2018). Autophagy Inhibitor (LY294002) and 5-Fluorouracil (5-FU) Combination-Based Nanoliposome for Enhanced Efficacy Against Esophageal Squamous Cell Carcinoma. Nanoscale Res. Lett..

[13] Liao Y., Guo Z., Xia X., Liu Y., Huang C., Jiang L., Wang X., Liu J., Huang H. (2019). Inhibition of EGFR Signaling with Spautin-1 Represents a Novel Therapeutics for Prostate Cancer. J. Exp. Clin. Cancer Res..

[14] Liu J., Xia H., Kim M., Xu L., Li Y., Zhang L., Cai Y., Vakifahmetoglu Norberg H., Zhang T., Furuya T. (2011). Beclin1 Controls the Levels of p53 by Regulating the Deubiquitination Activity of USP10 and USP13. Cell.

[15] Jang J., Jeung H.-K., Seol S.-Y., Chung H., Kim Y.R., Cheong J.-W., Min Y.H. (2018). Inhibition of Unc-51-Like Kinase 1 (ULK1) with Novel Small Molecular Inhibitor MRT68921 Preferentially Induces Apoptosis and Autophagy in FLT3-ITD-Mutated Acute Myeloid Leukemia. Blood.

[16] Avsec D., Jakoš Djordjevič A.T., Kandušer M., Podgornik H., Škerget M., Mlinarič-Raščan I. (2021). Targeting Autophagy Triggers Apoptosis and Complements the Action of Venetoclax in Chronic Lymphocytic Leukemia Cells. Cancers.

[17] Yuan N., Song L., Zhang S., Lin W., Cao Y., Xu F., Fang Y., Wang Z., Zhang H., Li X. (2015). Bafilomycin A1 Targets Both Autophagy and Apoptosis Pathways in Pediatric B-Cell Acute Lymphoblastic Leukemia. Haematologica.

[18] Fedele A.O., Proud C.G. (2020). Chloroquine and Bafilomycin A Mimic Lysosomal Storage Disorders and Impair mTORC1 Signalling. Biosci. Rep..

[19] Mauthe M., Orhon I., Rocchi C., Zhou X., Luhr M., Hijlkema K.J., Coppes R.P., Engedal N., Mari M., Reggiori F. (2018). Chloroquine Inhibits Autophagic Flux by Decreasing Autophagosome-Lysosome Fusion. Autophagy.

[20] Salata C., Calistri A., Parolin C., Baritussio A., Palù G. (2017). Antiviral Activity of Cationic Amphiphilic Drugs. Expert Rev. Anti-Infective Ther..

[21] Carew J.S., Espitia C.M., Esquivel J.A., Mahalingam D., Kelly K.R., Reddy G., Giles F.J., Nawrocki S.T. (2011). Lucanthone is a novel inhibitor of autophagy that induces cathepsin D-mediated apoptosis. J. Biol. Chem..

[22] Naidu M.D., Agarwal R., Pena L.A., Cunha L., Mezei M., Shen M., Wilson D.M., Liu Y., Sanchez Z., Chaudhary P. (2011). Lucanthone and Its Derivative Hycanthone Inhibit Apurinic Endonuclease-1 (APE1) by Direct Protein Binding. PLoS ONE.

[23] Jung M., Lee J., Seo H.-Y., Lim J.S., Kim E.K. (2015). Cathepsin Inhibition-Induced Lysosomal Dysfunction Enhances Pancreatic Beta-Cell Apoptosis in High Glucose. PLoS ONE.

[24] Mugume Y., Kazibwe Z., Bassham D.C. (2020). Target of Mycin in Control of Autophagy: Puppet Master and Signal Integrator. Int. J. Mol. Sci..

[25] Smolewski P. (2006). Recent Developments in Targeting the Mammalian Target of Rapamycin (mTOR) Kinase Pathway. Anti-Cancer Drugs.

[26] Niu T.-K., Pfeifer A.C., Lippincott-Schwartz J., Jackson C.L. (2005). Dynamics of GBF1, a Brefeldin A-Sensitive Arf1 Exchange Factor at the Golgi. Mol. Biol. Cell.

[27] Li D., Li M., Li X., Liu X., Gao W., Yu H., Bai J. (2022). Study of Brefeldin A on Induction of Autophagy in HepG2 Cells. Environ. Chem..

[28] Zhu K., Dunner K., McConkey D. (2010). Proteasome Inhibitors Activate Autophagy as a Cytoprotective Response in Human Prostate Cancer Cells. Oncogene.

[29] Liu J., Zhao R., Jiang X., Li Z., Zhang B. (2021). Progress on the Application of Bortezomib and Bortezomib-Based Nanoformulations. Biomolecules.

[30] Robak P., Robak T. (2019). Bortezomib for the Treatment of Hematologic Malignancies: 15 Years Later. Drugs R D.

[31] Cusack J.C., Liu R., Xia L., Chao T.H., Pien C., Niu W., Palombella V.J., Neuteboom S.T., Palladino M.A. (2006). NPI-0052 Enhances Tumoricidal Response to Conventional Cancer Therapy in a Colon Cancer Model. Clin. Cancer Res..

[32] Sarkar S., Rubinsztein D.C. (2006). Inositol and IP3 Levels Regulate Autophagy—Biology and Therapeutic Speculations. Autophagy.

[33] Chang J.-W., Choi H., Cotman S.L., Jung Y.-K. (2011). Lithium Rescues the Impaired Autophagy Process in CbCln3Δex7/8/Δex7/8 Cerebellar Cells and Reduces Neuronal Vulnerability to Cell Death via IMPase Inhibition. J. Neurochem..

[34] Vicencio J., Ortiz C., Criollo A., Jones A.W.E., Kepp O., Galluzzi L., Joza N., Vitale I., Morselli E., Tailler M. (2009). The Inositol 1,4,5-Trisphosphate Receptor Regulates Autophagy through Its Interaction with Beclin 1. Cell Death Differ..

[35] Kovács T., Billes V., Komlós M., Hotzi B., Manzéger A., Tarnóci A., Papp D., Szikszai F., Szinyákovics J., Rácz Á. (2017). The Small Molecule AUTEN-99 (Autophagy Enhancer-99) Prevents the Progression of Neurodegenerative Symptoms. Sci. Rep..

[36] Kovács T., Szinyákovics J., Billes V., Murányi G., Varga V.B., Bjelik A., Légrádi Á., Szabó M., Sándor S., Kubinyi E. (2022). A Conserved MTMR Lipid Phosphatase Increasingly Suppresses Autophagy in Brain Neurons during Aging. Sci. Rep..

[37] Zhu Z., Liu L.F., Su C.F., Liu J., Tong B.C.-K., Iyaswamy A., Krishnamoorthi S., Sreenivasmurthy S.G., Guan X.-J., Kan Y.-X. (2022). Corynoxine B Derivative CB6 Prevents Parkinsonian Toxicity in Mice by Inducing PIK3C3 Complex-Dependent Autophagy. Acta Pharmacol. Sin..

[38] Chen L.-L., Song J.-X., Lu J.-H., Yuan Z.-W., Liu L.-F., Durairajan S.S.K., Li M. (2014). Corynoxine, a Natural Autophagy Enhancer, Promotes the Clearance of Alpha-Synuclein via Akt/mTOR Pathway. J. Neuroimmune Pharmacol..

[39] Zhang P., Zheng Z., Ling L., Yang X., Zhang N., Wang X., Hu M., Xia Y., Ma Y., Yang H. (2017). w09, a Novel Autophagy Enhancer, Induces Autophagy-Dependent Cell Apoptosis via Activation of the EGFR-Mediated RAS-RAF1-MAP2K-MAPK1/3 Pathway. Autophagy.

[40] Briz V., Hsu Y.-T., Li Y., Lee E., Bi X., Baudry M. (2013). Calpain-2-Mediated PTEN Degradation Contributes to BDNF-Induced Stimulation of Dendritic Protein Synthesis. J. Neurosci..

[41] Shi M., Zhang T., Sun L., Luo Y., Liu D.-H., Xie S.-T., Song X.-Y., Wang G.-F., Chen X.-L., Zhou B.-C. (2013). Calpain, Atg5 and Bak Play Important Roles in the Crosstalk between Apoptosis and Autophagy Induced by Influx of Extracellular Calcium. Apoptosis.

[42] Khan H., Garg N., Singh T.G., Kaur A., Thapa K. (2022). Calpain Inhibitors as Potential Therapeutic Modulators in Neurodegenerative Diseases. Neurochem. Res..

[43] Ai J., Liu E., Wang J., Chen Y., Yu J., Baker A.J. (2007). Calpain Inhibitor MDL-28170 Reduces the Functional and Structural Deterioration of Corpus Callosum Following Fluid Percussion Injury. J. Neurotrauma.

[44] Çolak A., Karaoğlan A., Kaya M., Sağmanligil A., Akdemir O., Şahan E., Çelik Ö. (2009). Calpain Inhibitor AK 295 Inhibits Calpain-Induced Apoptosis and Improves Neurologic Function After Traumatic Spinal Cord Injury in Rats. Neurocirugía.

[45] Moldoveanu T., Campbell R.L., Cuerrier D., Davies P.L. (2004). Crystal Structures of Calpain–E64 and –Leupeptin Inhibitor Complexes Reveal Mobile Loops Gating the Active Site. J. Mol. Biol..

[46] Peng S., Kuang Z., Zhang Y., Xu H., Cheng Q. (2011). The Protective Effects and Potential Mechanism of Calpain Inhibitor Calpeptin Against Focal Cerebral Ischemia–Reperfusion Injury in Rats. Mol. Biol. Rep..

[47] Joudeh J., Claxton D. (2012). Obatoclax Mesylate: Pharmacology and Potential for Therapy of Hematological Neoplasms. Expert Opin. Investig. Drugs.

[48] Liang L.-Z., Ma B., Liang Y.-J., Liu H.-C., Zhang T.-H., Zheng G.S., Su Y.-X., Liao G.-Q. (2015). Obatoclax Induces Beclin 1- and ATG5-Dependent Apoptosis and Autophagy in Adenoid Cystic Carcinoma Cells. Oral Dis..

[49] Basit F., Cristofanon S., Fulda S. (2013). Obatoclax (GX15-070) Triggers Necroptosis by Promoting the Assembly of the Necrosome on Autophagosomal Membranes. Cell Death Differ..

[50] Koehler B.C., Jassowicz A., Scherr A.L., Lorenz S., Radhakrishnan P., Kautz N., Elssner C., Weiss J., Jaeger D., Schneider M. (2015). Pan-Bcl-2 Inhibitor Obatoclax is a Potent Late Stage Autophagy Inhibitor in Colorectal Cancer Cells Independent of Canonical Autophagy Signaling. BMC Cancer.

[51] Fulda S. (2017). Autophagy in Cancer Therapy. Front. Oncol..

[52] Lu G., Wu Z., Shang J., Xie Z., Chen C., Zhang C. (2021). The Effects of Metformin on Autophagy. Biomed. Pharmacother..

[53] Lim H., Lim Y.M., Kim K.H., Jeon Y.E., Park K., Kim J., Hwang H.Y., Lee D.J., Pagire H., Kwon H.J. (2018). A Novel Autophagy Enhancer as a Therapeutic Agent Against Metabolic Syndrome and Diabetes. Nat. Commun..

[54] Kim J., Park K., Kim M.J., Lim H., Kim K.H., Kim S.W., Lee E.S., Kim H., Kim S.J., Hur K.Y. (2021). An Autophagy Enhancer Ameliorates Diabetes of Human IAPP-Transgenic Mice Through Clearance of Amyloidogenic Oligomer. Nat. Commun..

